# Antibodies Recognizing *Yersinia enterocolitica* Lipopolysaccharides of Various Chemotypes in Synovial Fluids From Patients With Juvenile Idiopathic Arthritis

**DOI:** 10.1155/2022/9627934

**Published:** 2022-09-21

**Authors:** Katarzyna Kasperkiewicz, Anna S. Świerzko, Mateusz Michalski, Łukasz Eppa, Mikael Skurnik, Zbigniew Żuber, Maciej Cedzyński

**Affiliations:** ^1^Institute of Biology, Biotechnology, And Environmental Protection, Faculty of Natural Sciences, The University of Silesia in Katowice, Jagiellońska 28, 40-032 Katowice, Poland; ^2^Laboratory of Immunobiology of Infections, Institute of Medical Biology, Polish Academy of Sciences, Lodowa 106, PL 93-232 Łódź, Poland; ^3^Department of Bacteriology and Immunology, Medicum, Human Microbiome Research Program, Faculty of Medicine, University of Helsinki, Haartmaninkatu 3, 00290 Helsinki, Finland; ^4^Division of Clinical Microbiology, HUS LAB, University of Helsinki and Helsinki University Hospital, 00290 Helsinki, Finland; ^5^Pediatrics Clinic, Faculty of Medicine and Health Sciences, Andrzej Frycz Modrzewski Krakow University, Gustawa Herlinga-Grudzińskiego 1, 30-705 Kraków, Poland; ^6^Department of Rheumatology, St Louis Voivodeship Specialist Children's Hospital, Cracow, Poland

## Abstract

*Yersinia enterocolitica* O:3 (YeO3) is considered to be associated with reactive arthritis (ReA), and its lipopolysaccharide (LPS) has been detected in synovial fluids from patients. Interestingly, YeO3 wild-type LPS was processed by host cells, resulting in truncated LPS molecules presenting the core region. Previously, we reported the immunogenicity but not adjuvanticity of YeO3 LPSs of wild (S) type, Ra, Rd, or Re chemotypes in mice. Here, we demonstrate the presence of YeO3 LPS chemotype-specific antibodies in all analyzed synovial fluids (SF) from patients with juvenile idiopathic arthritis (JIA). Interestingly, the high titer of antibodies specific for the Kdo-lipid A region was found in most tested SF. In contrast, only a few were positive for antibodies recognizing O-specific polysaccharides. Western blot analysis revealed the presence of antibodies reacting with fast-migrating LPS fractions and enterobacterial common antigen (ECA) in synovial fluid samples. Our data also suggest the importance of LPS-associated ECA for the antigenicity of endotoxin. Furthermore, we confirmed *in vitro* that *Yersinia* LPS processing leads to the exposure of its core region and enhanced potency of complement lectin pathway activation.

## 1. Introduction

*Yersinia enterocolitica* O:3 (YeO3) is a causative agent of gastrointestinal infections but may also cause sepsis, with a mortality rate above 50% [1]. It is characterized by certain unique features like the ability to multiply at an extensive range of temperatures (from <4°C to >40°C) and by temperature-regulated expression of some virulence factors. Furthermore, in contrast to the majority of *Enterobacteriaceae*, its lipopolysaccharide (LPS, endotoxin) is composed of lipid A-inner core (IC) oligosaccharide backbone, substituted either with a long O-specific polysaccharide (OPS) chain (lipid A-IC-OPS) or an outer core oligosaccharide (OC) (lipid A-IC-OC) [2–5]. IC may be substituted with enterobacterial common antigen (ECA) polysaccharide (lipid A-IC-ECA-OPS) in OPS-carrying molecules, called ECA_LPS_. In most Gram-negative bacteria endotoxins, OPS is attached to the outer core (lipid A-IC-OC-OPS); therefore, the molecule contains both OC and OPS. The composition of YeO3 LPS is influenced by the temperature of growth. The lower favour the synthesis of molecules containing OPS while the higher culture temperature of bacteria those decorated by shorter OC. No experimental data concerning YeO3 LPS biosynthesis *in vivo* are available; however, both OPS and core region are essential for bacterial virulence. They modulate the activity of the LPS “toxic principle” (lipid A) and influence bacterial serum resistance. For example, shortening of OPS at 37°C is associated with the increased ability of *Yersinia* Ail factor (outer membrane protein) to bind inhibitors of classical, lectin, and alternative complement pathways (C4b-binding protein and H-factor) [6]. Additionally, substituting the inner core with OC or OPS/ECA prevents the interaction of mannose-binding lectin (MBL) interaction with IC heptose residues and associated complement lectin pathway activation, YeO3 endotoxin IC, OC, or OPS are receptors for bacteriophages, considered potential diagnostic and therapeutic agents [7].

In some cases of yersinioses, post-infection manifestations like erythema nodosum or myocarditis are observed. Moreover, as mentioned, *Y. enterocolitica* may cause sepsis, as a rare complication after blood transfusion (as it is able to survive in stored blood preparations) [8]. Infections caused by *Y. enterocolitica* strains of serotypes O:3 and O:9 are often complicated with the development of reactive arthritis (ReA) or juvenile idiopathic arthritis (JIA), mainly in HLA-B27 (human leukocyte antigen B27)-positive patients [9]. Although ReA was considered a sterile disease with no microbes found in the joints, immune complexes of *Yersinia* antigens, *Yersinia*-specific antibodies of IgM, IgG, and IgA, classes as well LPS were found in sera and synovial fluids from patients diagnosed with *Yersinia-*triggered ReA, even several years after infection [10]. We report here presence of *Yersinia* LPS-reactive antibodies, recognizing lipid A-Kdo, core oligosaccharide, or OPS in synovial fluids from patients suffering from JIA.

## 2. Materials and Methods

### 2.1. Clinical Material

Synovial fluid samples from 39 paediatric patients aged 3-18 years (mean 11.2 ± 0.1) were collected at the Department of Rheumatology, St Louis Voivodeship Specialist Children's Hospital (Cracow, Poland). Female patients accounted for 62%. Patients were subdivided into subgroups based on positive (JIA Ye+) or negative (JIA Ye-) results of the recomWell test (Mikrogen Diagnostics, Neuried, Germany), allowing for detection of IgG, IgM, and IgA antibodies and recognizing *Yersinia* outer membrane proteins (Yops) (Table 1). The results for 39 sera were available and reported earlier by Kasperkiewicz et al. [3]. Twelve of them (30.7%) were found to be positive. The study was approved by the Bioethics Committee of the Regional Medical Chamber in Cracow, and informed parental consent was obtained. This work confirms the provisions of the Declaration of Helsinki.

### 2.2. Bacterial Strains, Growth Conditions, and LPS Isolation

The bacteria used in this work are listed in Table 2. They have grown aerobically at 37°C in LB medium, in the presence of kanamycin or chloramphenicol when required. The LPS from smooth *Yersinia enterocolitica* O:3 (6471/76-c) and *Salmonella enterica* serovar Montevideo SH94 strains were isolated by the hot water method/water method [11]. In contrast, the LPS of the rough strains (YeO3-c-R1, YeO3-c-Rfb-R7, YeO3-c-R1-M205, and YeO3-c-OCR-ECA) were separated by the hot phenol/water extraction followed by the phenol/chloroform/petroleum ether method [12]. ECA from *S. enterica* serovar Montevideo SH94 as antigen was used to detect anti-ECA antibodies in sera and synovial fluids. ECA preparation was obtained thanks to Prof. Joanna Radziejewska-Lebrecht (University of Silesia).

### 2.3. Interaction of Mannose-Binding Lectin with LPS Exposed on Macrophages

The human monocytic THP-1 cell line came from the collection of the Laboratory of Immunobiology of Infections (IMB PAS, Łódź, Poland). The cells were routinely grown in RPMI-1640 (Gibco, USA) and supplemented with 10% of fetal bovine serum (FBS) (Sigma-Aldrich, USA), L-glutamine/streptomycin/penicillin, 1 mM pyruvate (Gibco, USA), and 50 mM *β*-mercaptoethanol (Gibco, USA). THP-1 monocytes (cultivated in eight-chamber slides) were differentiated into macrophages by 48 h incubation with PMA. Next, cells were fed with heat-killed bacteria. After 1 h of incubation, the cells were washed (4× with PBS) and further cultivated for 2 or 5 days. Next, the cells were fixed with PFA, blocked with 0.1% BSA, and incubated for 2 h at 37°C with normal human serum (NHS, Innovative Research, USA) as the source of MBL (prediluted in imidazole buffer, 40 mM C_3_H_4_N_2_, 1.25 M NaCl, 50 mM CaCl_2_, pH 7.8). After washing, the bound protein was detected with anti-human MBL mAb (clone HYB 131-01, Bioporto, Denmark) and Alexa-Fluor 488-labelled anti-mouse Ig (Invitrogen, USA). Nuclei were stained with DAPI, whereas actin was stained with Texas-Red-labelled phalloidin (Invitrogen, USA). In the case of negative control, the addition of NHS was omitted. The images were taken with the help of a confocal microscope (Nikon D-Eclipse C1 with EZ-C1 version 3.6 software, Japan).

### 2.4. SDS-PAGE and Western Blot

ECA from *Salmonella* Montevideo SH94 and LPS from YeO3 strains of various chemotypes were separated by SDS-PAGE (Mini-Protean apparatus; Bio-Rad, USA) [16]. As previously described, the products were transferred to PVDF membrane (Thermo Scientific, USA) and further processed for WB. After blocking, the membrane was incubated with synovial fluid (prediluted 1 : 150 in 1% BSA/TBS-Ca, 10 mM Tris, 140 mM NaCl, 1 mM CaCl_2_, pH 7.4) or corresponding patient serum (prediluted 1 : 300 in 1% BSA/TBS-Ca). The bound antibodies were detected with AP-labelled anti-human IgG, IgA, and IgM antibodies (Dako, Denmark), prediluted 1 : 2000 in Dot blot buffer (5 M NaCl, 1 M Tris, pH 7.4) with 10% of skim milk (Thermo Fisher Scientific, United Kingdom). AP-conjugated antibodies were detected with 5-bromo-4-chloro-3-indolyl-phosphate (BICIP) and p-nitro blue tetrazolium chloride (NBT) (Serva, Germany) in AP-buffer. To exclude nonspecific binding of secondary antibody to LPS, membrane was incubated with AP-labelled antibody only.

### 2.5. Detection of Antibodies Reactive Against *Yersinia* LPS

NUNC Maxisorp U96 (Denmark) plates were coated with tested LPS (5 *μ*g/ml in PBS). After blocking with 1% BSA, synovial fluid samples (prediluted 1 : 50 in 1% BSA/TBS-Ca) were added. After 2 h of incubation at 37°C, the bound antibodies were detected with HRP-labelled rabbit anti-human IgG, IgA, and IgM antibodies (Dako). ABTS (Sigma) was used as the substrate for peroxidase. The OD values were normalized against BSA-coated well. The reaction was estimated as positive if the OD value was at least twice higher than the negative control.

### 2.6. Statistical Analysis

GraphPad Prism 9 software (GraphPad, USA) was used for statistical analysis. *p*values < 0.05 were considered statistically significant.

## 3. Results

### 3.1. Detection of Antibodies Recognizing *Yersinia enterocolitica* Lipopolysaccharides of Various Chemotypes

Clinical material was taken during the active phase of the disease from two male patients (16 and 14 years old), strongly positive (patient I) and weakly positive (patient II) for *Yersinia* Yops-specific IgG, IgA, and IgM antibodies [3]. Patients were diagnosed 4 and 2.3 months earlier, with three or > five affected joints, respectively.

**Figure 1:**
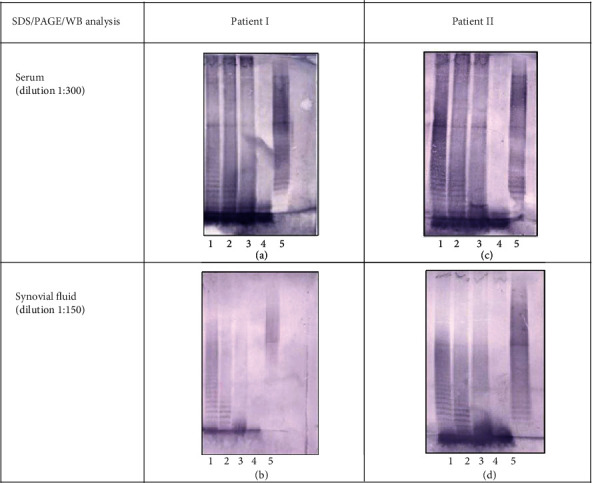
Detection of *Yersinia* LPS- and ECA- reactive antibodies in sera and synovial fluids from patients I and II. Wild (S) type, Ra, Rc and Re chemotype LPS and ECA preparations were separated in SDS-PAGE. After transfer to PVDF, the membranes were incubated with synovial fluid or corresponding patient serum. Lanes 1-YeO3-c (wild-type) LPS, 2-YeO3-c-R1 (Ra) LPS, 3-YeO3-c-Rfb-R7 (Rc) LPS, 4-YeO3-c-M205LPS (Re) LPS, and 5-*S.* Montevideo SH94 ECA.

As mentioned above, *Yersinia enterocolitica* lipopolysaccharide-reactive antibodies were previously detected in synovial fluids from patients diagnosed with ReA [18]. We not only confirmed this finding in JIA but also demonstrated that those Ab can recognize wild-chemotype YeO3-c-6471/76 LPS as well as lipopolysaccharides of Ra (YeO3-c-R1), Rc (YeO3-c-RfbR7), and deep-rough mutant Re (YeO3-c-M205) chemotypes (Figure 1). Interestingly, the detected antibodies recognized the fast-migrating LPS fraction, corresponding to the lipid-A-inner core LPS fraction. Additionally, the ladder-like pattern observed for YeO-c-R1 LPS (Figures 1(a)–1(d), lane 2) may suggest that antibodies recognize ECA attached to LPS (ECA_LPS_) in sera and synovial fluids are present. It was also confirmed by detecting ECA-recognizing antibodies using ECA preparation from *S.* Montevideo SH94. The recognition pattern in synovial fluid (Figures 1(b) and 1(d)) was a weaker reflection of antibodies detected in the corresponding patient's sera (Figures 1(a) and 1(c)), likely suggesting that the SF antibodies originate from blood. The reactivity of antibodies present in synovial fluids of both patients with *Y. enterocolitica* LPS preparations was also similar despite a more intense signal observed for patient II. No reaction with LPS or ECA was observed when pooled normal serum (see the supplementary materials, Figure 1 A) or secondary antibodies alone were used (see the supplementary materials, Figure 1 B).

### 3.2. LPS Processing by Macrophages Leads to LPS Core Region Exposition

**Figure 2:**
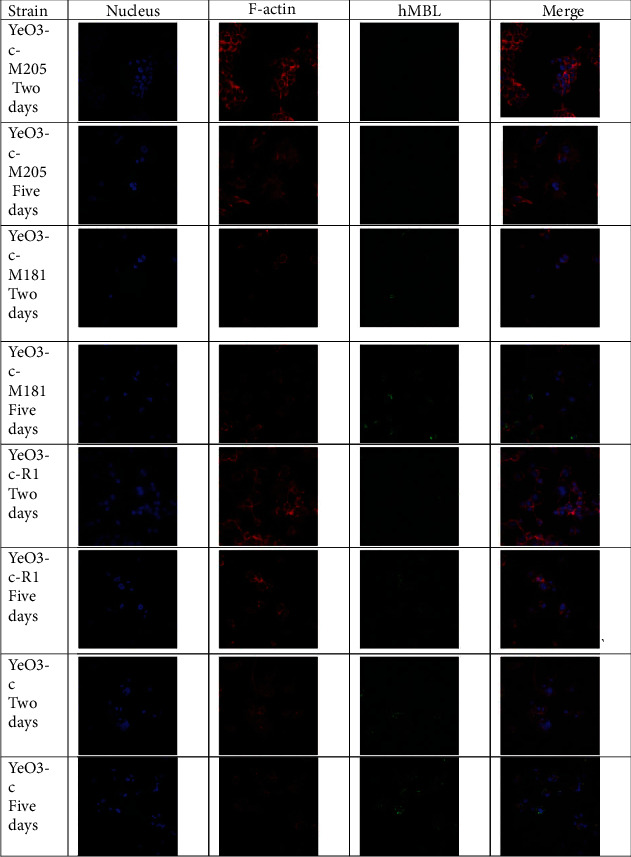
*In vitro* processing of *Yersinia* LPS. PMA differentiated THP-1 cells were fed with heat-killed bacteria of various chemotypes. After 2 or 5 days of cultivation, cells were fixed with PFA and incubated with human serum as a source of MBL. The cell-surface bound MBL was detected with an MBL-specific monoclonal antibody and Alexa Fluor 488 labelled secondary antibody.

To test expected *Yersinia enterocolitica* LPS processing by phagocytes, THP-1 cells were differentiated into macrophages and fed with heat-killed bacteria of wild-type (YeO3-c), Ra (YeO3-c-R1), Rd (YeO3-c-M181), or Re (YeO3-c-M205) chemotypes. According to our previous results, the OPS and outer core oligosaccharide degradation and the exposure of the inner core were detected via analysis of MBL binding [3]. YeO3-c-M181 (Rd chemotype) bacteria were positive, whereas YeO3-c-M205 was a negative control. Obtained data demonstrated that prolonged cultivation of macrophages with wild-type or Ra chemotype bacteria led to increased MBL binding (suggesting the exposure of LPS inner core due to LPS intracellular processing) (Figure 2). In contrast, no binding was detected for macrophages fed with Re chemotype (YeO3-c-M205) cells, devoid of inner core heptoses.

### 3.3. Determination of Specificity of *Yersinia* LPS-Reactive Antibodies Present in Synovial Fluids

Next, we analyzed the presence and specificity of antibodies recognizing *Yersinia* O:3 LPS in synovial fluids from children/teenagers diagnosed with JIA. To detect antibodies recognizing OPS, LPS OC core, LPS IC, or Kdo-lipid A backbone LPS from *Yersinia enterocolitica* wild-type strain and Ra and Re LPS chemotype mutants were used as plate-coating antigens in ELISA. Based on the results, synovial fluid samples analyzed could be classified into five groups:

*Group I*. Synovial fluids positive for antibodies recognizing Kdo-lipid A region but negative for antibodies reacting with YeO3-c, YeO3-c-R1, and YeO3-c-OCR-ECA lipopolysaccharides.

*Group II*. Synovial fluids positive for antibodies recognizing Kdo-lipid A region as well as OC but negative for antibodies reacting with YeO3-c, YeO3-c-Rfb-R7, and YeO3-c-OCR-ECA LPSs.

*Group III*. Synovial fluids positive for antibodies recognizing Kdo-lipid A region, OC, and IC but negative for antibodies reacting with YeO3-c and YeO3-c-OCR-ECA lipopolysaccharides.

*Group IV*. Synovial fluids positive for antibodies recognizing Kdo-lipid A region and OPS and OC and partially positive for the IC but negative for antibodies recognizing IC not substituted with ECA in YeO3-c-OCR-ECA LPS.

*Group V*. Synovial fluids positive for antibodies recognizing Kdo-lipid A region as well as OC and partially recognizing IC substituted or not substituted with ECA.

**Figure 3:**
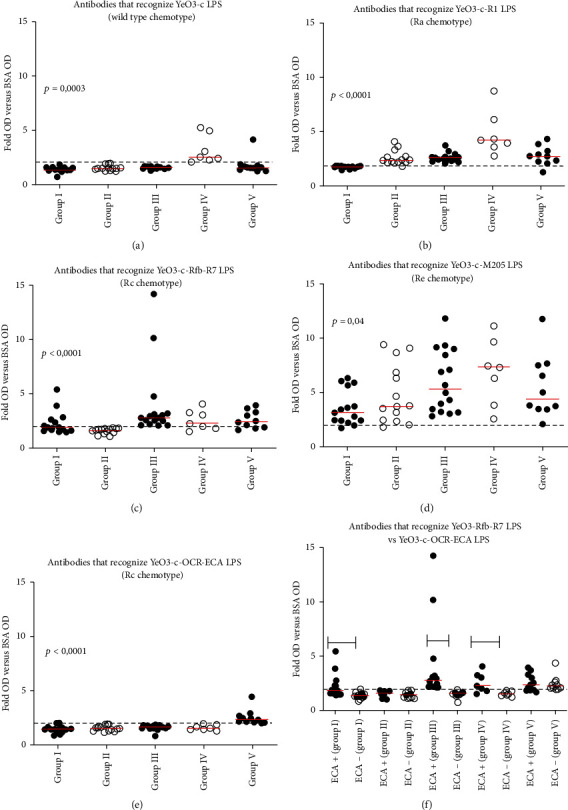
Reactivity of synovial fluid antibodies with LPSs: YeO3-c (wild-type chemotype) (a), YeO3-c-R1 (Ra chemotype) (b), YeO3-c-Rfb-R7 (Rc chemotype) (c), YeO3-c-M205 (Re chemotype) (d), and YeO3-c-OCR-ECA (Rc chemotype without ECA) (e) in groups I-V. Comparison of the levels of ECA-dependent (YeO3-c-Rfb-R7, ECA+) and ECA-independent (YeO3-c-OCR-ECA, ECA-) antibodies between groups (f). Red bars represent median values. One-way ANOVA or Mann–Whitney *U* test was used for statistical analysis. Horizontal black lines show the statistically significant differences between the analyzed groups (*p* < 0.05, see details within the text), while one-way ANOVA results are given when significant.

To summarize, we found the presence of *Yersinia*-reactive antibodies in all analyzed synovial fluid samples (Figure 3). Interestingly, in almost all tested SF, high levels of antibodies recognizing deep-rough mutant YeO3-c-M205 LPS were observed (Figure 3(d)). In contrast, antibodies specific for wild-type LPS were detected in group IV only (Figure 3(a)). OC-reactive antibodies were found in II, III, IV and V groups (Figure 3(b)) whereas antibodies directed against the IC substituted with ECA - in I, III, IV and V groups (Figure 3(c)). In group IV, synovial fluids contain the mixture of antibodies specific for *Y. enterocolitica* OPS, OC, and IC oligosaccharides, and deep IC region (Figures 3(a)–3(d)). Antibodies for which the presence of ECA seemed not to be essential were found in group V only (Figure 3(e)). The importance of substituting *Yersinia* LPS with ECA for antibody induction was supported by the fact that 39 synovial fluids were positive for YeO3-c-Rfb-R7 LPS (with ECA attached to the LPS) and only 9 for YeO3-c-OCR-ECA (without ECA related to the LPS), both representing Rc chemotype. Moreover, the levels of Rc-specific ECA-dependent antibodies in groups I, III, and IV were significantly higher than in ECA-independent Ab (Figure 3(f); *p* = 0.0001; *p* < 0.0001, and *p* = 0.0111, respectively).

## 4. Discussion

The incidence and prevalence of JIA are estimated as 5-18 and 30-150/100 000 children, respectively [18]. In Poland, 5-6 new cases per 100 000 individuals are diagnosed yearly [19]. Several JIA subtypes were defined based on clinical features: systemic arthritis, oligoarthritis, polyarthritis, enthesitis-related arthritis, psoriatic arthritis, and undifferentiated arthritis. To be diagnosed with JIA, the patient at disease onset should be <16 years old, with a minimum duration of symptoms of 6 weeks [18]. Many other childhood arthropathies have been described, including infection-associated septic arthritis, reactive arthritis, acute rheumatic fever, and irritable hip [18].

Paediatric ReA is currently classified as enthesitis-related JIA, as proposed by the International League of Associations for Rheumatology (ILAR) [20]. ReA, a type of spondylarthritis, is usually mono- or oligoarthritis, often associated with extra-articular manifestations (genitourinary, skin, ophthalmologic, musculoskeletal, and cardiovascular) [20]. Usually, it refers to inflammatory disease in the absence of microorganisms in the joint but connected with an extra-articular infection, mainly in the gastrointestinal or urogenital tract. It may reflect disturbances of immune tolerance due to recurrent or latent infections, molecular mimicry, or the effect of heat shock proteins, and superantigens [21].

*Yersinia enterocolitica* (O:3 and O:9 serotypes mainly) is one the most common agents involved in the pathogenesis of reactive arthritis after gastrointestinal infections. In Poland, YeO3 is also the most common causative agent of yersiniosis [19]. *Yersinia* LPS or ECA-specific antibodies were detected in 22% and 35% of synovial fluid specimens from Polish patients with spondyloarthropathies and rheumatoid arthritis [19]. Moreover, SF contained Ye-specific immune complexes [22] and antibodies against *Yersinia* LPS [23]. Although the titer of antibodies was lower in synovial fluids, it correlated significantly with their serum levels. Fotis et al. [24] suggested that the increased concentration of antibodies directed against the R3-type core region of *E. coli* LPS in the circulation of paediatric patients with JIA is associated with increased intestinal bacterial translocation and circulating LPS. Moreover, Aoki et al. [25] demonstrated the presence of antibodies reacting with ECA-substituted LPS (ECA_LPS_) in sera and synovial fluids from adult patients who have rheumatoid arthritis (RA).

Recently, we demonstrated that coinjection of collagen and lipopolysaccharide is not associated with the increase of anti-collagen antibody production in mice, excluding the involvement of adjuvant activity of LPS during experimental RA development [26]. Surprisingly, *Yersinia* LPS injection decreased anti-collagen antibody production (although insignificantly). However, LPSs of all tested chemotypes were found to be potent immunogens themselves [26].

The origin of LPS in the synovial fluid has not been established. Since all attempts to grow *Yersinia* from the synovial fluids have failed, LPS may be transported to joints by inflammatory cells. In line with this hypothesis, Granfors et al. [27] demonstrated that the synovial fluid mono- and polymorphonuclear cells from patients with reactive arthritis associated with *Yersinia* infection contain endotoxin [9]. Moreover, Wuorela et al. [10] evidenced that LPS molecules may undergo intracellular processing, resulting in the presentation of core oligosaccharides on the phagocyte surface. We demonstrated that the prolonged culture of macrophages fed with heat-killed *Yersinia* wild-type and Ra chemotype bacteria increased MBL binding by surface-exposed bacterial antigens (Figure 2). It suggests that LPS processed by macrophages results in the presentation of IC of LPS, which we showed previously to be a target for MBL [3].

We also confirmed the presence of *Yersinia* LPS-reacting antibodies in synovial fluids from patients diagnosed with JIA (Figure 3). Moreover, we demonstrated that those Ab might recognize epitopes located in OPS and the OC and/or IC oligosaccharides (Figure 3). We also showed that ECA-reactive antibodies are present in synovial fluid, and ECA is essential for antibody induction (Figure 3(f)). Previously, ECA-specific antibodies were detected in sera of >30% of Polish patients diagnosed with rheumatoid arthritis [28]. As mentioned earlier, the *Yersinia* IC region was shown to be substituted with ECA, which could induce the production of specific antibodies [4, 29]. Interestingly, most Ab existing in synovial fluid recognize LPS of Re chemotype (Figure 3(d)). However, it may result from the cross-reactivity of possibly present in joint antibodies against the conserved Kdo-lipid A region, typical for many Gram-negative bacteria. However, we cannot exclude that YeO3-c-M205 LPS preparation coats Maxisorp plate wells more efficiently than LPS preparations containing more extended polysaccharide components. Moreover, we also found a negative association of anti-YeO3-c-M205 antibodies with CRP. In the group of patients with no antibodies against OPS and IC and no ECA-independent immunoglobulins, the trend toward highest values of ESR, CRP, and longer disease duration was observed (data not shown).

## 5. Conclusions

We demonstrated the presence of antibodies recognizing *Yersinia* LPS in all analyzed synovial fluids obtained from patients suffering from JIA with confirmed *Yersinia* infection and from JIA patients with no detectable antibodies against Yops in serum. Most antibodies were directed against the Kdo-lipid A region of LPS, and antibodies against OPS were detected only in a few synovial fluids samples. It may suggest the better usability of LPS preparations from *Yersinia* LPS rough mutants in developing new assays to detect prolonged infections with those bacteria. It was also confirmed by demonstrating exposure of the IC region in LPS processing by phagocytes. Moreover, our results also suggest the significance of ECA presence for inducing LPS-specific antibodies.

## Figures and Tables

**Table 1:** The comparison of clinical data for JIA patients positive and negative for *Yersinia* Yops antibodies.

	JIA patients		Statistical analysis
	No Yops antibodies were detected in the serum (Ye-)	Yops antibodies were detected in serum (Ye+)	
Number of patients (*N*)	27	12	—
Age (mean)	11.1	11.2	*p* > 0.05∗
Male/female	7/20	5/7	*p* > 0.05∗∗
Anti-nuclear antibodies (ANA) (number of patients)	22	9	*p* > 0.05∗∗
Number of affected joints mean/median	2.7/2	2.6/2	*p* > 0.05∗∗
Disease duration (months) mean/median	4.1/3.2	5.7/4.2	*p* > 0.05∗
Erythrocyte sedimentation rate (ESR) mean/median	11.8/7	5.2/5.5	*p* < 0.05∗
C-reactive protein (CRP) mean/median	8.9/4.9	5.9/4.9	*p* < 0.05∗

∗: Mann–Withney *U* test; ∗∗: Fisher's exact test.

**Table 2:** Bacterial strains used in this work for isolation of LPS (c-cured of virulence plasmid pYV^−^; R-O-antigen missing).

Strain	Description	LPS chemotype	References
*Yersinia enterocolitica* 6471/76-c (YeO3-c)	Virulence plasmid cured derivative of 6471/76	S (LA-IC-OPS) and Ra (LA-IC-OC)	[13]
*Yersinia enterocolitica* YeO3-c-R1	Spontaneous rough derivative of strain 6471/76-c; pYV−	Ra (LA-IC-OC)	[2]
*Yersinia enterocolitica* YeO3-c-Rfb- R7	Tn5-phoA insertion mutant in OC gene cluster, pYV−, KmR	Rc (LA-IC)	[14, 15]
*Yersinia enterocolitica* YeO3-c-OCR-ECA	YeO3-c-OCR (wzzE-wzyE) ECA gene cluster deleted. pYV−, KmR.	Rc (LA-IC)	[15]
*Yersinia enterocolitica* YeO3-c-R1-M181	YeO3-c-R1 *galU*::cat-Mu, clmR; pYV−	Rd1 (LA-4/8IC)	[4]
*Yersinia enterocolitica* YeO3-c-R1-M205	YeO3-c-R1 hldE::cat-Mu, clmR; pYV−	Re (LA-2/8IC)	[4]

## Data Availability

Data are available on request from the corresponding author.

## Supplementary Materials

Supplementary Materials Supplementary Figure 1 A: Detection of *Yersinia* LPS- and ECA- reactive antibodies in normal pooled human serum (Innovative Research, USA). Supplementary Figure 1 B: Cross-reactivity of secondary antibodies with Yersinia LPS and ECA.
