# Supplementary material for: Antibodies Recognizing Yersinia enterocolitica Lipopolysaccharides of Various Chemotypes in Synovial Fluids From Patients With Juvenile Idiopathic Arthritis
Source: J Immunol Res. 2022 Sep 21;2022:9627934. doi: 10.1155/2022/9627934 (PMC9519298; doi:10.1155/2022/9627934)
Supplement: Supplementary Materials — Supplementary Figure 1 A: Detection of Yersinia LPS- and ECA- reactive antibodies in normal pooled human serum (Innovative Research, USA). Supplementary Figure 1 B: Cross-reactivity of secondary antibodies with Yersinia LPS and ECA. [file 9627934.f1.docx]

**Supplementary materials**


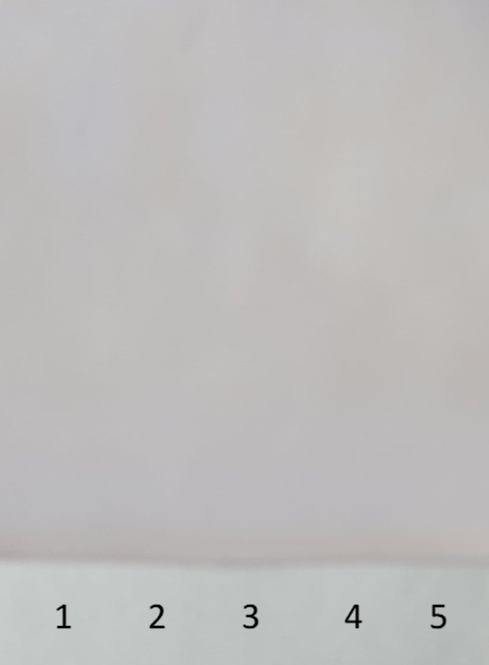


**Supplementary** **Figure 1 A**: Detection of *Yersinia* LPS- and ECA- reactive antibodies in normal pooled human serum (Innovative Research, USA).

Wild (S) type, Ra-, Rc- and Re-chemotype LPS and ECA preparations were separated in SDS-PAGE. After transfer to PVDF, the membranes were incubated with tested serum
 (1:300 in 1% BSA/TBS-Ca) and with alkaline phosphatase labeled monoclonal antibodies against human immunoglobulins (Dako, Denmark).

Lanes: 1-YeO3-c (wild type) LPS; 2-YeO3-c-R1 (Ra) LPS; 3-YeO3-c-Rfb-R7 (Rc) LPS; 4-YeO3-c-M205LPS (Re) LPS; 5 - *S.* Montevideo SH94 ECA.


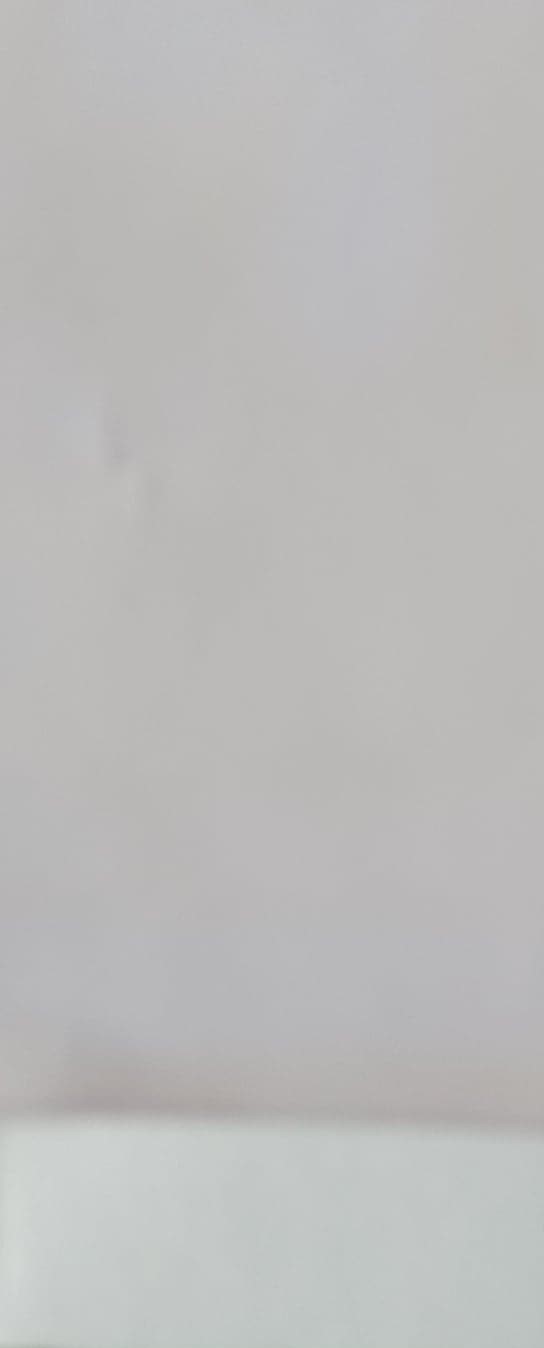


1 2 3 4 5

**Supplementary Figure 1 B**: Cross-reactivity of secondary antibodies with *Yersinia* LPS
and ECA.

Wild (S) type, Ra-, Rc- and Re-chemotype LPS and ECA preparations were separated in SDS-PAGE. After transfer to PVDF, the membranes were incubated with monoclonal antibodies against human immunoglobulins labeled with alkaline phosphatase (Dako, Denmark) (serum addition was omitted). Lanes: 1-YeO3-c (wild type) LPS; 2-YeO3-c-R1 (Ra) LPS; 3-YeO3-c-Rfb-R7 (Rc) LPS; 4-YeO3-c-M205LPS (Re) LPS; 5 - *S.* Montevideo SH94 ECA.
